# Vericiguat Modulates Osteoclast Differentiation and Bone Resorption via a Balance between VASP and NF-*κ*B Pathways

**DOI:** 10.1155/2022/1625290

**Published:** 2022-06-16

**Authors:** Kaiqiang Sun, Fanqi Kong, Feng Lin, Fudong Li, Jingchuan Sun, Changzhen Ren, Bing Zheng, Jiangang Shi

**Affiliations:** ^1^Department of Orthopedic Surgery, Spine Center, Changzheng Hospital, Naval Medical University, No. 415 Fengyang Road, Shanghai, 200003, China; ^2^Department of Orthopedic Surgery, The Affiliated Yantai Yuhuangding Hospital of Qingdao University, Shandong, China; ^3^Department of General Practice, The 960th Hospital of PLA, Jinan, China

## Abstract

Bone homeostasis has been a dynamic equilibrium between osteoclasts (OCs) and osteoblasts (OBs). However, excessive activation of OCs could disturb the bone homeostasis. As a result, effective medical interventions for patients are greatly demanding. NO/guanylate cyclase (GC)/cGMP signaling cascade has been previously reported to regulate bone metabolism, and GC plays a significantly critical role. Vericiguat, as a novel oral soluble guanylate cyclase (sGC) stimulator, has been firstly reported in 2020 to treat patients with heart failure. Nevertheless, the biological effects of Vericiguat on the function of OCs have not yet been explored. In this present study, we found that Vericiguat with the concentration between 0 and 8 *μ*M was noncytotoxic to bone marrow-derived monocyte-macrophage lineage (BMMs). Vericiguat could enhance the differentiation of OCs at concentration of 500 nM, whereas it inhibited OC differentiation at 8 *μ*M. In addition, Vericiguat also showed dual effects on OC fusion and bone resorption in a dose-dependent manner. Furthermore, a molecular assay suggested that the dual regulatory effects of Vericiguat on OCs were mediated by the bidirectional activation of the I*κ*B-*α*/NF-*κ*B signaling pathway. Taken together, our present study demonstrated the dual effects of Vericiguat on the formation of functional OCs. The regulatory effects of Vericiguat on OCs were achieved by the bidirectional modulation of the I*κ*B-*α*/NF-*κ*B signaling pathway, and a potential balance between the I*κ*B-*α*/NF-*κ*B signaling pathway and sGC/cGMP/VASP may exist.

## 1. Introduction

Bone tissue consistently undergoes dynamic changes, by means of new bone formation by osteoblasts (OBs) and aging bone elimination by osteoclasts (OCs) [1, 2]. On the contrary, excessive activation of OCs will trigger a disequilibrium between OCs and OBs and thus cause osteolytic bone diseases, such as osteoporosis [3]. Collectively, agents targeting the suppression of the activation of OCs are still clinically demanding.

Osteoclastogenesis is significantly necessary for bone resorption. Normally, OCs are differentiated from bone marrow-derived monocyte-macrophage lineage (BMMs), under the stimulation of macrophage colony-stimulating factor (MCSF) and receptor activator of nuclear factor-*κ* B ligand (RANKL) [3, 4]. MCSF combined with RANKL will further activate downstream signaling pathways, mitogen-activated protein kinases (MAPK), NF-*κ*B pathways, and PI3K/AKT pathways [5, 6]. Then, the activated pathways can activate nuclear factor of activated T cell cytosolic 1 (NFATc1) and c-Fos. These two factors induce the expression of OC-specific markers, including tartrate resistant acid phosphatase (TRAP), cathepsin K (CTSK), calcitonin receptor (CTR), and dendritic cell-specific transmembrane protein (DC-STAMP) [7]. Thus, mature OCs will produce a tight sealing zone via the formation of F-actin and secrete related enzymes into its sealing zone to accomplish the bone resorption [7]. As a result, agents targeting RANKL/RANK signaling cascades are effective strategy to treat OC-related bone diseases.

Previous studies have suggested that NO is widely involved in regulating bone metabolism via directly activating guanylate cyclase (GC). Rangaswami et al. ever reported that mechanical stimulation could induce OB proliferation via NO/sGC/cGMP(cGMP) signaling cascades [8]. Furthermore, NO was demonstrated to regulate the mobility and detachment of OCs in a concentration-dependent manner via cGMP [9]. Nevertheless, the activated oxidative stress mediated by NO has limited its clinical application [10]. Therefore, regulating the activity of cellular GC may provide a novel perspective into the treatment of osteoporosis. In fact, animal experiment by Joshua et al. has suggested that treatment with soluble GC (sGC) activators could attenuate estrogen deficiency-induced bone loss [11]. A recent study reported that pharmacologically activating sGC in an NO- or heme-independent manner could be a new therapeutic strategy to suppress cementum loss [12]. Vericiguat, a novel oral soluble guanylate cyclase (sGC) stimulator, has been firstly reported to reduce the death incidence from cardiovascular causes or hospitalization for heart failure patients in 2020 [13, 14]. However, the effects of Vericiguat on the function of OCs remain elusive.

Therefore, this present study was aimed at investigating the biological effects of Vericiguat on OC differentiation and potential molecular mechanisms, which will provide novel reference for future treatment of bone loss-related diseases.

## 2. Materials and Methods

All animal experiments in this study complied with the ARRIVE guidelines and were carried out in accordance with the National Research Council's Guide for the Care and Use of Laboratory Animals.

### 2.1. Bone Marrow-Derived Macrophage (BMM) Isolation

This method has been described in detail in our previous study [15]. Briefly, the whole bone marrow fluid was flushed from the mice femurs and tibias using a sterile 5 ml syringe with complete *α*-MEM (30 ng/ml M-CSF) and then grown in a T-25 cm^2^ flask for 5 days. Adherent cells were BMMs. The harvested BMMs were used for the subsequent experiments.

### 2.2. Cell Viability Assay

This method has been described in detail in our previous study [15]. Vericiguat used in this present study was purchased from MedChemExpress (HY-16774). The cytotoxicity of Vericiguat on BMMs was examined by using the CCK-8 assay kit (Dojindo Institute of Biotechnology, Kumamoto, Japan). Briefly, BMMs were seeded in a 96-well plate (4-5 × 10^3^ cells/well) for 24 hours. Then, BMMs were added with Vericiguat (0, 10, 20, 50, 100, 200, and 500 nM and 1, 2, 4, 8, and 10 *μ*M) for another 24 hours. Subsequently, BMMs were incubated with 100 *μ*l CCK-8 test solution in a 37°C incubator for 2-3 h. The absorbance was detected at a wavelength of 450 nm.

### 2.3. OC Differentiation Assay In Vitro

BMMs were cultured with density of 10000 cells/well in a 96-well plate. RANKL (50 ng/ml) without or with Vericiguat (0, 100 *μ*M, 500 nM, 1 *μ*M, 2 *μ*M, 4 *μ*M, and 8 *μ*M) was added at the second day for 6 d. Recombinant mouse-derived M-CSF and RANKL were purchased from Novoprotein Scientific Inc. (Pudong New District, China). The identification of OC was evaluated by TRAP staining using a tartrate resistant acid phosphatase staining kit (Sigma-Aldrich Institute of Biotechnology, St. Louis, MO, USA). TRAP-positive cells were observed and imaged using light microscopy (Nikon, Tokyo, Japan).

### 2.4. F-actin Ring Assay and Bone Resorption Pit Assay

These two methods have been described in detail in our previous study [15]. Briefly, matured OCs were fixed using 4% paraformaldehyde for 10 minutes, followed by permeabilization in 0.1% Triton X-100 for another 10 minutes and then incubation with phalloidin diluted solution (G1028, Servicebio, Wuhan, China) for 2 h in the dark. Nuclei were stained with DAPI staining. The F-actin belt was detected using fluorescence microscopy.

For the bone resorption assay, after BMMs fused, an equal number of fused OCs were transplanted onto Corning Osteo Assay Surface Multiple Well Plate (Corning, Inc., Corning, NY, USA). After OC adhesion, the cells were treated with 0 and 500 nM and 8 *μ*M Vericiguat for 2-3 days. Subsequently, the cells on the plates were eliminated. The percentage of the resorbed region was randomly measured in totally three resorption sites from three independent experiments using ImageJ (National Institutes of Health, Bethesda, MD).

### 2.5. Real-Time Quantitative PCR (qRT-PCR)

This method has been described in detail in our previous study [15]. Total cellular RNA was harvested using the RNA extraction kit (Magen, Inc., Guangzhou, China). The acquired mRNA was then reversed using HiScript® III RT SuperMix for qPCR Kit (R323-01, Vazyme, Nanjing, China). Subsequently, the mRNA expression levels were detected by qRT-PCR using the SYBR qPCR Master Mix (Q711-02, Vazyme, Nanjing, China). The reaction conditions were reported in our previous study as follows: one cycle at 95°C for 30 s (step 1), followed by 40 cycles at 95°C for 10 s and at 60°C for 30 s (step 2). All experiments were repeated for 3 times, and GAPDH was used as the internal reference. The primer sequences used for qRT-PCR analysis are shown in Table 1.

### 2.6. Western Blot

To investigate the effects of Vericiguat on osteoclastogenesis-related signaling pathways, pre-OCs were pretreated with *α*-MEM (nonserum and M-CSF) for 2 hours followed by Vericiguat (0, 100 nM, 200 nM, 500 nM, 2 *μ*M, and 8 *μ*M) for 3-4 hours, and then, BMM cells were incubated with RANKL (50 ng/ml) for another 30 minutes. In terms of the protein expression level of c-Fos and NFATc1, RANKL (50 ng/ml) with Vericiguat (0, 100 nM, 500 nM, 1 *μ*M, 2 *μ*M, 4 *μ*M, 6 *μ*M, and 8 *μ*M) was added to BMMs for 3 days. In addition, to confirm the effects of Vericiguat (500 nM and 8 *μ*M) on the protein expression of NFATc1 and c-Fos, we also evaluated the cytoplasmic and nuclear protein expression of NFATc1 for 0, 2, and 4 days, respectively.

The method of protein extraction has been described in detail in our previous study [15]. Briefly, BMMs were lysed in RIPA on the ice, and the protein amount was quantified using the protein measurement kit (Beyotime Institute of Technology, Shanghai, China). A total of 20-30 *μ*g protein per lane was separated by means of electrophoresis (10% sodium dodecyl sulphate polyacrylamide gel, 80 V, 110 min). Subsequently, the isolated protein bands were transferred onto the polyvinylidene fluoride membrane (EMD Millipore, Billerica, MA, USA) for 100 minutes, with the voltage of 100 V. Next, blocking was conducted by using 5% nonfat milk (Invitrogen, San Diego, CA, USA) dissolving in Tris-buffered saline-Tween for 3 hours at appropriately 28°C. Then, the PVDF membranes were washed for three times (5 min per time) using TBST. The membranes were then immersed in specific primary antibodies at 4°C for one night. On the second day, the membranes were submerged in the secondary antibodies (goat against rabbit or mouse, 1 : 1000-5000) for 2-3 hours at appropriately 28°C. Finally, the protein bands were captured using the Imaging System (version 5200, Tanon Science & Technology Co., Ltd., Shanghai, China).

Specific primary antibodies in this study mainly included Bax (342772, 17 kDa; Zenbio, Chengdu, China, 1 : 500), Bcl-2 (250198, 26 kDa; Zenbio, Chengdu, China, 1 : 500), NF-*κ*B p65 (D14E12, #8242, Cell Signaling Technology, Inc., 3 Trask Lane, Danvers, USA), p-p65 (Ser536; #3033, Cell Signaling Technology, Inc., 3 Trask Lane, Danvers, USA), Ik-Ba (380682, 35 kDa; Zenbio, Chengdu, China, 1 : 1,000), p-Ik-Ba (340776, 35 kDa; Zenbio, Chengdu, China, 1 : 1,000), ERK1/2 (201245-4A4, 42/44 kDa; Zenbio, Chengdu, China, 1 : 1,000), p-ERK1/2 (301245, 42/44 kDa; Zenbio, Chengdu, China, 1 : 1,000), p38 (200782, 43 kDa; Zenbio, Chengdu, China, 1 : 500), p-p38 (310069, 43 kDa; Zenbio, Chengdu, China, 1 : 1,000), JNK (381100, 46/54 kDa; Zenbio, Chengdu, China, 1 : 1,000), p-JNK (380556, 46/54 kDa; Zenbio, Chengdu, China, 1 : 1,000), c-Fos (9F6, #2250, Cell Signaling Technology, Inc., 3 Trask Lane, Danvers, USA), NFATc1 (#8032; D15F1, Cell Signaling Technology, Inc., 3 Trask Lane, Danvers, USA), AKT (#4691; D15F1, Cell Signaling Technology, Inc., 3 Trask Lane, Danvers, USA), p-AKT (#4060; D15F1, Cell Signaling Technology, Inc., 3 Trask Lane, Danvers, USA), mTOR (380411, 289 kDa; Zenbio, Chengdu, China, 1 : 500), p-mTOR (381548, 289 kDa; Zenbio, Chengdu, China, 1 : 500), GAPDH (#5174, D16H11, Cell Signaling Technology, Inc., 3 Trask Lane, Danvers, USA), and histone H3 (250182, 15 kDa; Zenbio, Chengdu, China, 1 : 1,000).

### 2.7. Nuclear Translocation of the NF-*κ*B p65 Assay

This method has been described in detail in our previous study [15]. Briefly, BMMs were incubated with *α*-MEM (without serum and M-CSF) for 2 hours, and then, the wells were added with Vericiguat (0, 500 nM, and 8 *μ*M) for 3 hours, followed by stimulation with or without RANKL (50 ng/ml) for 30 minutes. Subsequently, the BMMs were fixed for 30 minutes using 4% paraformaldehyde and permeabilized for another 30 minutes in 0.1% Triton X-100. Next, cells were immerged in nonfat milk (5%) in PBST for 2-3 hours, followed by incubation with the p65 primary antibody for one night at 4°C. Finally, cells were added with FITC-conjugated goat anti-rabbit IgG (Servicebio, Wuhan, China) for 50 minutes, and then, the nucleus was stained with DAPI (Sigma; St. Louis, MO, USA) for 5 minutes. Cells were washed three times using PBS and imaged using a laser scanning confocal microscope.

### 2.8. Transfection of si-VASP

The siRNA of VASP was designed and purchased from GenePharma (Shanghai, China). si-RNA was transfected into cells by means of Lipofectamine 3000 (Thermo Fisher Scientific, USA). The expression of mRNA and protein was evaluated after 24 h and 48 h, respectively. The highest efficiency for knockdown of VASP was as follows: forward (GAGCCAAACUCAGGAAAGUTT) and reverse (ACUUUCCUGAGUUUGGCUCTT).

### 2.9. Molecular Modeling Experiments

Molecular modeling experiments were performed using the Schrӧdinger Maestro 9.0 package. Receptor docking (Glide), combined with the protein structure prediction, was used to analyze ligand and receptor flexibility based on the program Prime. In order to prepare protein, the program Protein Preparation Wizard was used to prepare the IKK*β* cocrystal structure by means of the Schrödinger Maestro suite prior to performing docking experiments. Water molecules were firstly removed from the crystal structure, followed by adding the hydrogen atoms, and then, the resultant structure was refined using OPLS_2005 force field. The minimization ended with hydrogens. Subsequently, the Receptor Grid Preparation option present in Glide was utilized to produce the protein grid, and the protein grid was subsequently applied to docking experiments. The van der Waals radius scaling factor was set to 1.0 (the partial charge cutoff was 0.25). The ligand was prepared with LigPrep in Maestro 9.0 and the OPLS_2005 force field. Epika was used to produce possible states at target pH 7.00 ± 3.00. Ligand docking options were used in Glide for the first round of docking experiments. In terms of setting, XP (extra precision), “dock flexibly,” “sample nitrogen inversions,” “sample ring conformation,” and “Epik state penalties” were selected to the docking score.

### 2.10. Establishment of the Ovariectomy- (OVX-) Induced Bone Loss Model In Vivo and Evaluation of the Therapeutic Effects of Vericiguat

This procedure has been reported in our previous study [15]. According to the study design, the C57BL/6 was randomly divided into four groups (*n* = 5): sham group (mock operation with DMSO injection), OVX group (OVX with DMSO injection), low-dose group (OVX with low-dose Vericiguat, 5 *μ*g/kg/day), and high-dose group (OVX with high-dose Vericiguat, 10 *μ*g/kg/day). After ending the experiment, the femurs were collected for microcomputed tomography (micro-CT) analysis [15]. In addition, after *μ*CT analysis, the bone samples were further decalcified in EDTA solution (10%) for another 4 weeks and then embedded in paraffin blocks for subsequent sectioning. Sections were then stained with hematoxylin eosin or TRAP staining. The area and the number of OCs were quantified using the ImageJ software (National Institutes of Health, Bethesda, MD) [15].

## 3. Statistical Analysis

Statistical analyses were carried out by means of GraphPad Prism 8 (GraphPad Software Inc.; La Jolla, CA). The quantified data were presented as mean ± standard deviation. All experiments were repeated independently at least for three times. One-way analysis of variance (ANOVA) was performed, followed by the Student–Neuman–Keuls as the post hoc test. A *p* value < 0.05 was considered to indicate statistical difference.

## 4. Results

### 4.1. Cytotoxicity Effect of Vericiguat on BMMs

**Figure 1:**
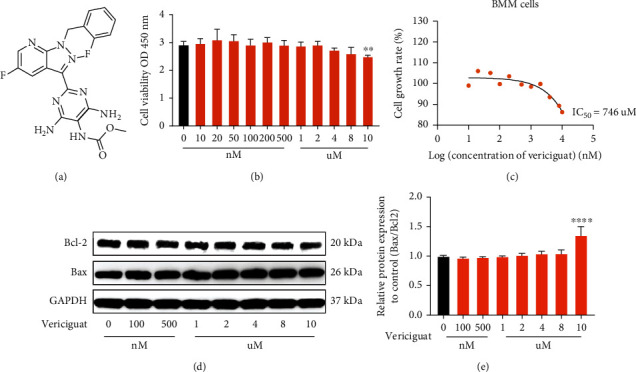
Molecular structure and cytotoxicity of Vericiguat on bone marrow-derived monocyte-macrophage lineage (BMMs). (a) Molecular structure of Vericiguat; (b) BMM cell viability as detected by CCK-8 assay after treatment with indicated concentrations of Vericiguat for 24 h. Data are presented as mean ± standard deviation (*n* = 5). (c) The IC_50_ values of Vericiguat against BMMs. (d, e) The effect of Vericiguat on apoptosis-related markers, Bax and Bcl-2, in BMMs and quantitative results (*n* = 3). ∗ indicated a comparison with the control group (0 nM): ^∗^*p* < 0.05, ^∗∗^*p* < 0.01, ^∗∗∗^*p* < 0.001, and ^∗∗∗∗^*p* < 0.0001.

Figure 1 shows the chemical structure of Vericiguat (Figure 1(a)). The cell viability was detected using the CCK-8 viability assay, and we found that Vericiguat below 8 *μ*M showed no cytotoxicity to BMMs, with the IC_50_ being 746 *μ*M (Figures 1(b) and 1(c)). In addition, we evaluated the expression of apoptosis-related markers, Bax and Bcl-2, and found similar results to those of the CCK-8 assay (Figures 1(d) and 1(e)). The results above indicated that the concentration range of Vericiguat between 0 and 8 *μ*M was noncytotoxic to BMMs.

### 4.2. Dual Effects of Vericiguat on RANKL-Induced Osteoclastogenesis In Vitro

**Figure 2:**
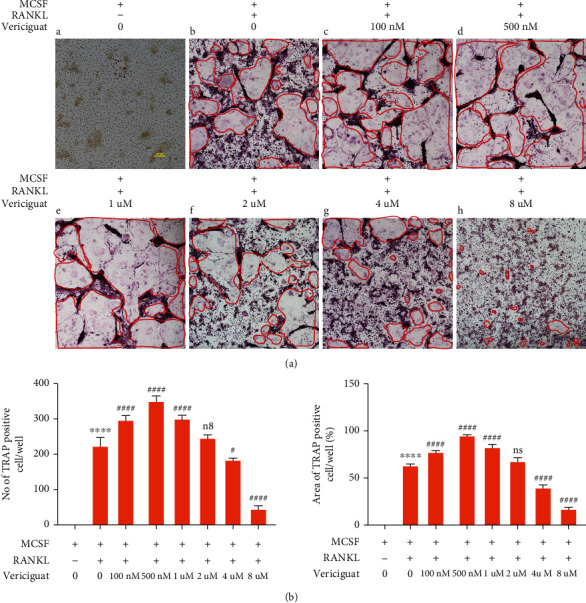
Vericiguat dually regulated RANKL-triggered osteoclast formation in vitro. (a) Representative images of TRAP-positive osteoclasts (red circles) after stimulation with RANKL and indicated concentrations of Vericiguat (*n* = 3). Image was used as the negative control (BMMS without RANKL). (b) The number and the size of TRAP^+^ multinucleated osteoclasts were quantified. *n* = 3 means three independent experiments. ∗ indicated a comparison with the control group (without RANKL and Vericiguat): ^∗^*p* < 0.05, ^∗∗^*p* < 0.01, ^∗∗∗^*p* < 0.001, and ^∗∗∗∗^*p* < 0.0001. # indicated a comparison between the group with RANKL only and the group with RANKL and Vericiguat: ^#^*p* < 0.05, ^##^*p* < 0.01, ^###^*p* < 0.001, and ^####^*p* < 0.0001. ns: not statistically significant. Scar bar = 100 *μ*m.

According to the results above, we explored the effects of Vericiguat on RANKL-induced functional OCs in vitro. As shown in Figure 2(a), Vericiguat enhanced differentiation of OCs at concentrations of 100 nM, 500 nM, and 1 *μ*M, but it inhibited differentiation at 4 *μ*M and 8 *μ*M in terms of the number and size of OCs (Figures 2(a) and 2(b)).

These results above indicated that Vericiguat showed dual effects on RANKL-induced osteoclastogenesis in vitro.

### 4.3. Vericiguat Dually Regulated RANKL-Mediated OC Formation and Bony Resorption in a Concentration-Dependent Manner

**Figure 3:**
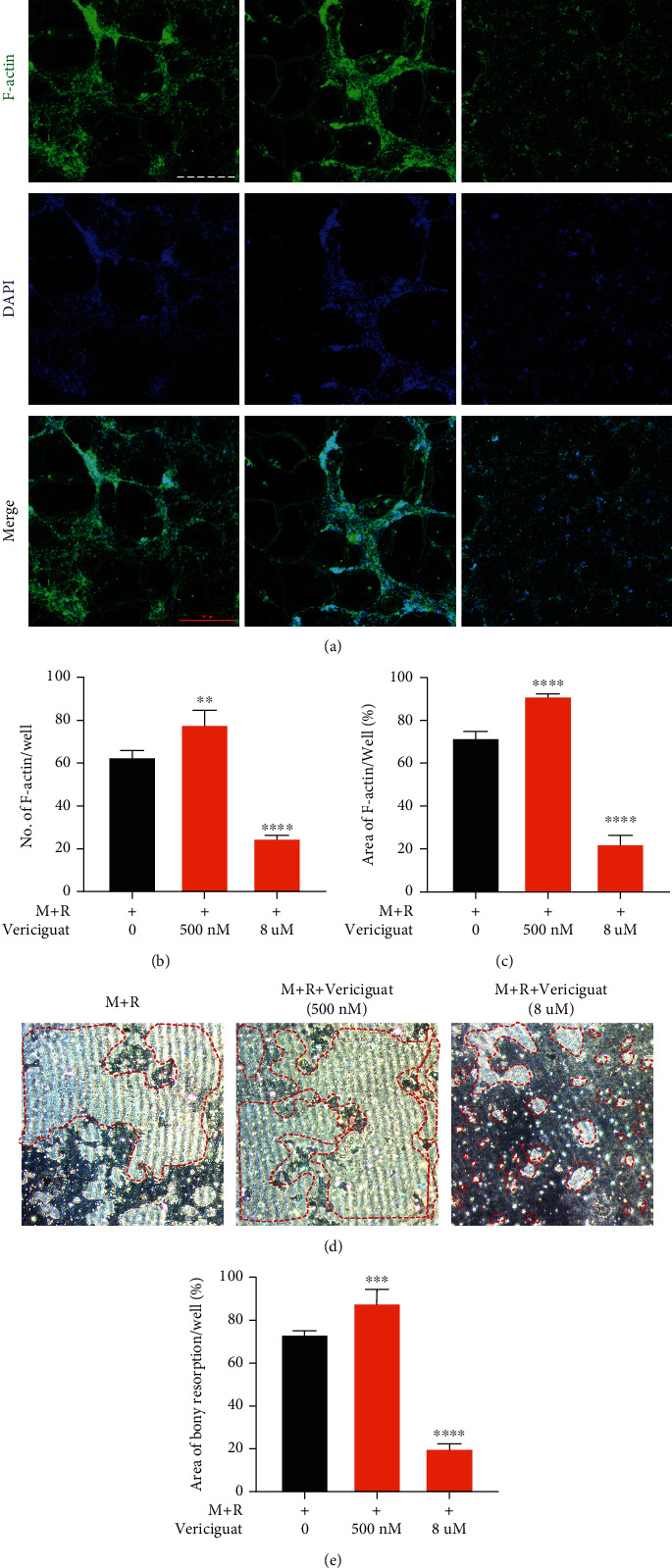
Vericiguat dually regulated the formation of F-actin ring and bony resorption of osteoclasts. (a) Representative images of F-actin-stained osteoclasts under the stimulation of RANKL for 7 d with 500 nM and 8 *μ*M Vericiguat, respectively. (b) The average number and area of F-actin ring (c) (*n* = 3). (d) Representative images of bony resorption (white area circled by a red dotted line) by mature osteoclasts. (e) The bony resorption area was quantified. Values are presented as the mean ± standard deviation (*n* = 3). ∗ indicated a comparison with the control group (Vericiguat, 0 nM): ^∗^*p* < 0.05, ^∗∗^*p* < 0.01, ^∗∗∗^*p* < 0.001, and ^∗∗∗∗^*p* < 0.0001. Scale bar = 500 *μ*m.

The formation of the F-actin belt has been the most typical feature of mature OCs [15]. As shown in Figure 3, mature OCs have a typical F-actin ring (Figure 3(a)). However, the F-actin belt surrounding the OCs was increased under low concentration of Vericiguat (500 nM) but decreased when the concentration of Vericiguat was high (8 *μ*M) (Figure 3(a)). The number and size of the F-actin belt also showed similar tendency to the FITC-phalloidin staining (Figures 3(b) and 3(c)).

In addition, we explored whether Vericiguat could affect the bony resorption of OCs. BMMs were grown onto the plate (M-CSF 30 ng/ml, RANKL 50 ng/ml) with Vericiguat of different concentrations. As suggested in Figure 3, the area of bone resorption was expanded under Vericiguat at low concentration (500 nM) but decreased when the concentration of Vericiguat was high (8 *μ*M) (Figures 3(d) and 3(e)).

Collectively, Vericiguat showed dual effects on the RANKL-induced formation of the F-actin belt and bony resorption in a concentration-dependent manner.

### 4.4. Vericiguat Dually Regulated RANKL-Mediated OC Marker Gene Expression during Osteoclastogenesis

**Figure 4:**
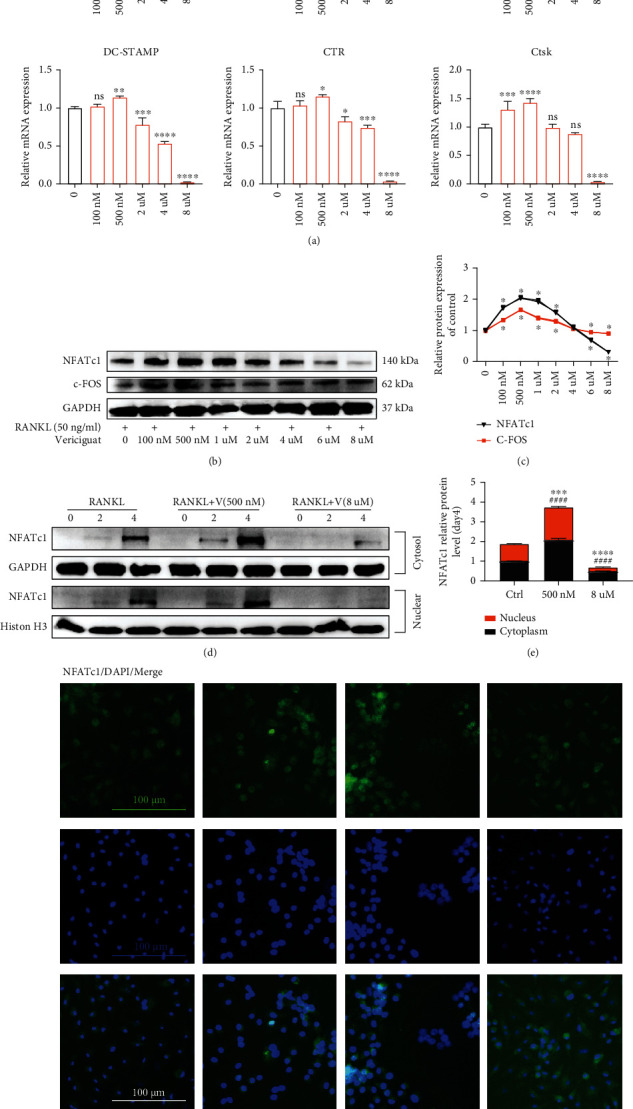
Vericiguat dually regulated RANKL-triggered osteoclast-specific gene expression in osteoclast formation. (a) The relative mRNA expression of osteoclast marker genes (c-Fos, NFATc1, TRAP, CTSK, CTR, and DC-STAMP) in BMMs treated with RANKL+MCSF, as well as indicated concentrations of Vericiguat, for three days was quantified by qRT-PCR (*n* = 3). Values are presented as the mean ± standard deviation. ∗ indicated a comparison with the control group (Vericiguat, 0 nM): ^∗^*p* < 0.05, ^∗∗^*p* < 0.01, ^∗∗∗^*p* < 0.001, and ∗∗∗∗*p* < 0.0001. (b, c) Western blot analysis results on the protein expression levels of OC-related markers including c-Fos and NFATc1 in BMM cells stimulated with RANKL for 5 d without or with Vericiguat with indicated concentrations and quantitative results (*n* = 3). ∗ indicated a comparison with the control group (Vericiguat, 0 nM): ^∗^*p* < 0.05, ^∗∗^*p* < 0.01, ^∗∗∗^*p* < 0.001, and ^∗∗∗∗^*p* < 0.0001. (d, e) The cytoplasmic and nuclear fractions of the BMMs treated with 30 ng/mL M-CSF, 50 ng/mL RANKL, and Vericiguat (500 nM and 8 *μ*M) for 0, 2, and 4 d, respectively, were analyzed by western blotting and quantitative results. GAPDH and histone H3 were used as nuclear and cytoplasmic loading controls, respectively (*n* = 3). ∗ indicated a comparison with the control group (Vericiguat, 0 nM) regarding the protein expression of NFATc1 within the cytosol at day 4 after stimulation: ^∗∗∗^*p* < 0.001 and ^∗∗∗∗^*p* < 0.0001. # indicated a comparison with the control group (Vericiguat, 0 nM) regarding the protein expression of NFATc1 within the nucleus at day 4 after stimulation: ^####^*p* < 0.0001. (f) Immunofluorescence results of the nuclear translocation of NFATc1 at day 4 after stimulation showed that Vericiguat at low concentration promoted the nuclear translocation of NFATc1, whereas high-concentration Vericiguat suppressed this effect. ns: not statistically significant. Scale bar = 100 *μ*m.

After treatment with Vericiguat (0, 100 nM, 500 nM, 2 *μ*M, 4 *μ*M, and 8 *μ*M), the mRNA expression of OC marker genes was markedly upregulated in the low-concentration group (100 nM, 500 nM, and 2 *μ*M) during osteoclastogenesis, including c-Fos, NFATc1, TRAP, DC-STAMP, CTR, and CTSK. On the contrary, the mRNA expression of those genes was significantly downregulated in the high-concentration group (4 *μ*M and 8 *μ*M) (Figure 4(a)).

RANKL leads to the induction and activation of NFATc1 and c-Fos, the two critical factors for osteoclastogenesis [16]. Resultantly, we explored the effects of Vericiguat on the protein expression of these two markers. Western blot results revealed that the expression of these two markers was enhanced at low concentration (100 nM-2 *μ*M) but decreased at high concentration (8 *μ*M), consistent with the results of qRT-PCR above (Figures 4(b) and 4(c)). NFATc1 nuclear translocation exerts a critical role in the regulation of the transcription of OC-related genes, such as DC-STAMP, TRAP, CTR, and CTSK [17]. Western blot suggested that RANKL triggered NFATc1 translocation into the nucleus during OC differentiation in a time-dependent manner (Figures 4(d) and 4(e)). Vericiguat promoted the RANKL-induced nuclear translocation of NFATc1 in the low-concentration group (500 nM), whereas this effect was inhibited in the high-concentration group (8 *μ*M) (Figures 4(d) and 4(e)). Immunofluorescence results of the nuclear translocation of NFATc1 at day 4 after stimulation showed similar tendency to the results of Western blot (Figure 4(f)).

Therefore, these results confirmed the dual effects of Vericiguat on RANKL-induced osteoclastogenesis in vitro.

### 4.5. Vericiguat Dually Regulated RANKL-Induced Activation of the NF-*κ*B Signaling Cascade

**Figure 5:**
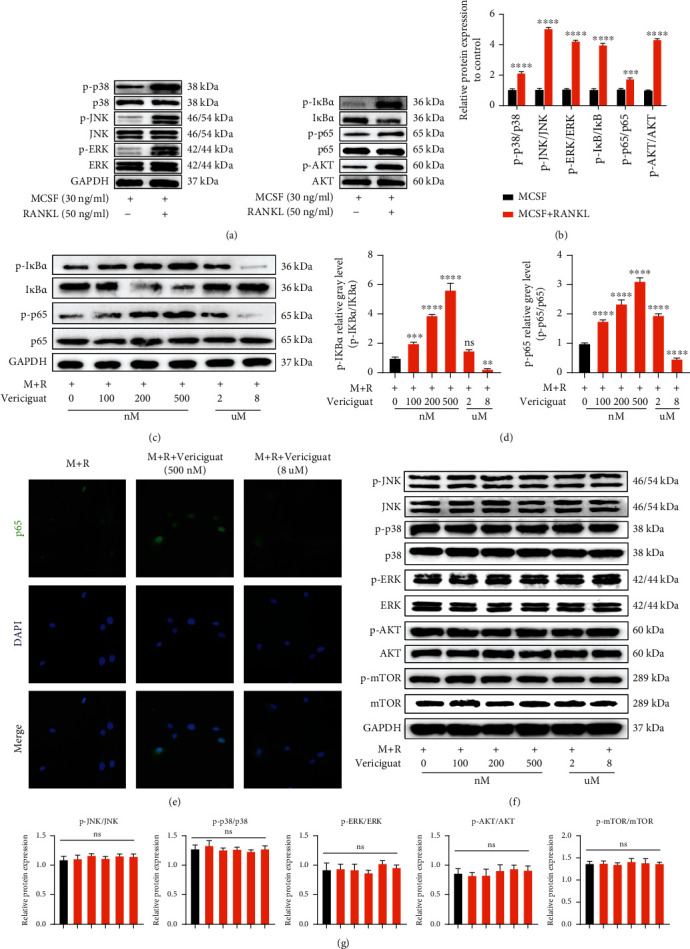
Vericiguat dually regulated RANKL-triggered activation of I*κ*B-*α*/NF-*κ*B signaling pathway. (a, b) Western blot results of the expression of osteoclast-related signaling pathways (NF-*κ*B, AKT, and MAPK) with or without RANKL stimulation (*n* = 3). ∗ indicated a comparison with the control group without RANKL. (c, d) Western blot results of the expression of I*κ*B-*α* and NF-*κ*B in BMMs after treatment with RANKL (50 ng/ml) for 30 min followed by Vericiguat with indicated concentrations. (e) Immunofluorescence staining of RANKL-induced P65 nuclear translocation with or without Vericiguat with indicated concentrations. (f, g) Western Blot and quantified results of MAPK and AKT in BMMs after treatment with RANKL (50 ng/ml) for 30 min followed by Vericiguat with indicated concentrations. *n* = 3 means three independent experiments. ∗ indicated a comparison with the control group (Vericiguat, 0 nM): ^∗^*p* < 0.05, ^∗∗^*p* < 0.01, ^∗∗∗^*p* < 0.001, and ^∗∗∗∗^*p* < 0.0001. ns: not statistically significant. Scale bar = 100 *μ*m.

As shown in Figure 5, RANKL significantly induced the activation of the NF-*κ*B signaling pathway, MAPK signaling pathways (p38, JNK, and ERK), and AKT signaling pathway (Figures 5(a) and 5(b)). To identify the changes of those signaling pathways during OC development after stimulation with Vericiguat, we examined the activation of those critical pathways. As demonstrated in Figure 5, the activation of the I*κ*B-*α*/p65 signaling pathway was dually regulated by Vericiguat in a concentration-dependent manner. The phosphorylation of I*κ*B-*α* and p65 was enhanced in the low-concentration group (100 nM-2 *μ*M) but decreased at high concentration (8 *μ*M) (Figures 5(c) and 5(d)). In addition, the early nucleus translocation of p65 after RANKL stimulation with or without Vericiguat as determined by the immune-fluorescent staining suggested an increasing trend at low concentration and a decreasing trend at high concentration (Figure 5(e)). However, the MAPK (p38, JNK, and ERK) signaling pathway and AKT signaling pathway were not affected by Vericiguat stimulation (Figures 5(f) and 5(g)).

Taken together, the results above suggested that Vericiguat was involved in OC differentiation via dually regulating the activation of the I*κ*B-*α*/NF-*κ*B signaling pathway.

### 4.6. VASP Was Essential to OC Differentiation, and the Expression of VASP Could Be Promoted by Vericiguat

**Figure 6:**
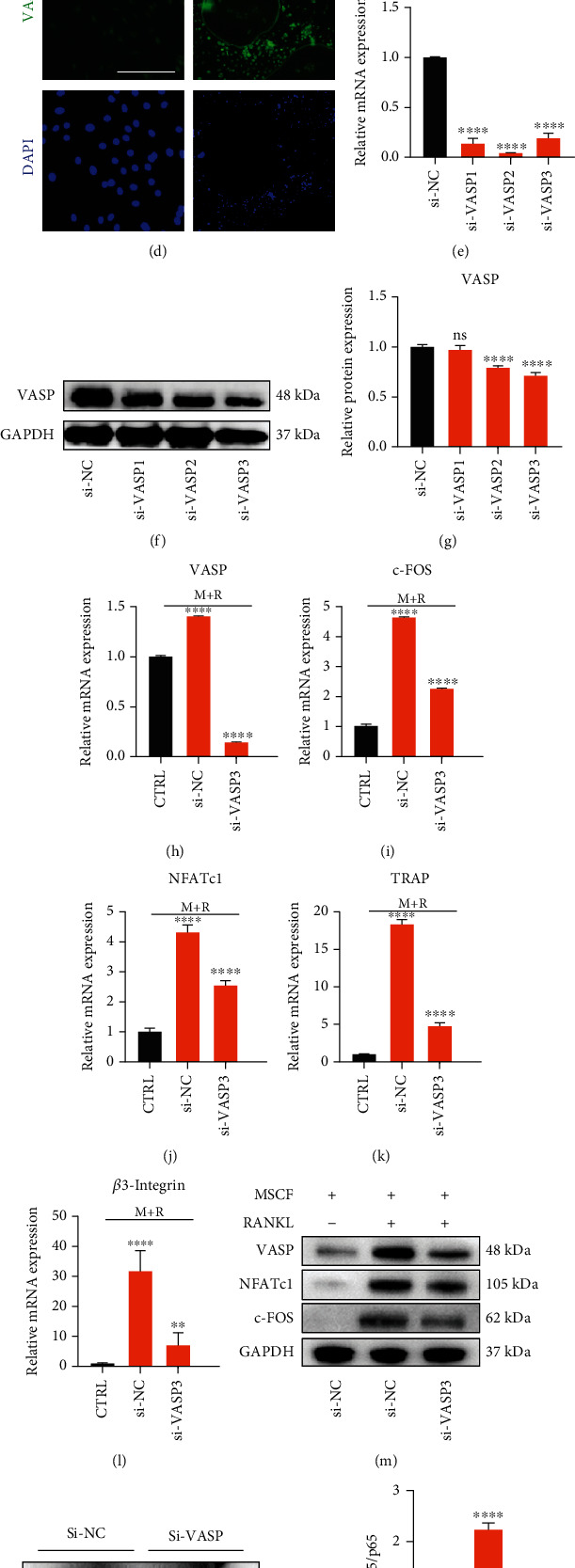
VASP was essential to osteoclast differentiation, and the expression of VASP could be promoted by Vericiguat. (a) VEGT augmented the expression of VASP in a dosage-dependent manner (*n* = 3). (b, c) RANKL enhanced the expression of VASP in a dosage-dependent manner and quantitative results (*n* = 3). ∗ indicated a comparison with the control group without RANKL. (d) Immunofluorescence position of VASP in BMMs with or without RANKL. (e) The mRNA expression of VASP in BMMs after treatment with siRNA (*n* = 3); ∗ indicated a comparison with the control group (si-NC). (f, g) The protein expression of VASP in BMMs after treatment with siRNA and quantitative results (*n* = 3). ∗ indicated a comparison with the control group (si-NC). (h–l) The mRNA expression of OC-related genes in BMMs after treatment with siRNA (*n* = 3). ∗ indicated a comparison with the control group: ^∗^*p* < 0.05, ^∗∗^*p* < 0.01, ^∗∗∗^*p* < 0.001, and ^∗∗∗∗^*p* < 0.0001. # indicated a comparison between the group with si-VASP and the group with si-NC: ^#^*p* < 0.05, ^##^*p* < 0.01, ^###^*p* < 0.001, and ^####^*p* < 0.0001. (m) The protein expression of osteoclast-related genes in BMMs after treatment with siRNA and quantitative results (*n* = 3). (n, o) The expression of NF-*κ*B protein in BMMs with or without siRNA-VASP treated with Vericiguat only (*n* = 3). (p) The mRNA expression of osteoclast-related genes (NFATc1, TRAP, and *β*3-Integrin) in siRNA-VASP-induced BMMs compared with siRNA-NC-induced BMMs (*n* = 3); ∗ indicated a comparison with the control group (Vericiguat, 0 nM) in the siRNA-NC group and si-VASP group, respectively: ^∗^*p* < 0.05, ^∗∗^*p* < 0.01, ^∗∗∗^*p* < 0.001, and ^∗∗∗∗^*p* < 0.0001. # indicated a comparison between the siNC-induced group and si-VASP-induced group at the same concentration of Vericiguat: ^#^*p* < 0.05, ^##^*p* < 0.01, ^###^*p* < 0.001, and ^####^*p* < 0.0001. Scale bar = 100 *μ*m.

The activated sGC would excessively induce the activation of the VASP (vasodilator-stimulated phosphoprotein) [18]. Therefore, we evaluated the expression of VASP in BMMs and found the increasing expression of VASP induced by a dose-dependent manner of Vericiguat with RANKL (Figure 6(a)). In order to investigate the potential dually regulative effects of Vericiguat on OC differentiation, we firstly evaluated the expression of VASP in BMMs induced by RANKL and found that RANKL could dose-dependently increase the expression of VASP (Figures 6(b) and 6(c)). In addition, mature OC also highly expressed VASP, and VASP protein was primarily distributed surrounding the cell membrane (Figure 6(d)). However, OC differentiation was significantly disturbed after silencing the expression of VASP via siRNA in BMMs, as shown by downregulated expression of OC-related marker genes (c-FOS, NFATc1, TRAP, and *β*3-integrin) (Figures 6(e)–6(m)). The results above suggested that VASP was essential to OC differentiation. However, the promotive effect of Vericiguat only on the amount of p-p65 was blocked by the silencing of VASP (Figures 6(n) and 6(o)). Notably, in the presence of MCSF and RANKL, after silencing of VASP, the promotive effect of Vericiguat (500 *μ*M) on OC differentiation was also blocked, whereas the suppressive effect of Vericiguat (8 *μ*M) on osteoclast differentiation was enhanced (Figure 6(p)). The results above suggested that VASP could mediate the promotive effect of Vericiguat on the phosphorylation of p65 under low concentration with or without the presence of MCSF and RANKL.

### 4.7. Dual Bioeffects of Vericiguat on OC Formation and Bony Resorption via a Balance between VASP and NF-*κ*B

**Figure 7:**
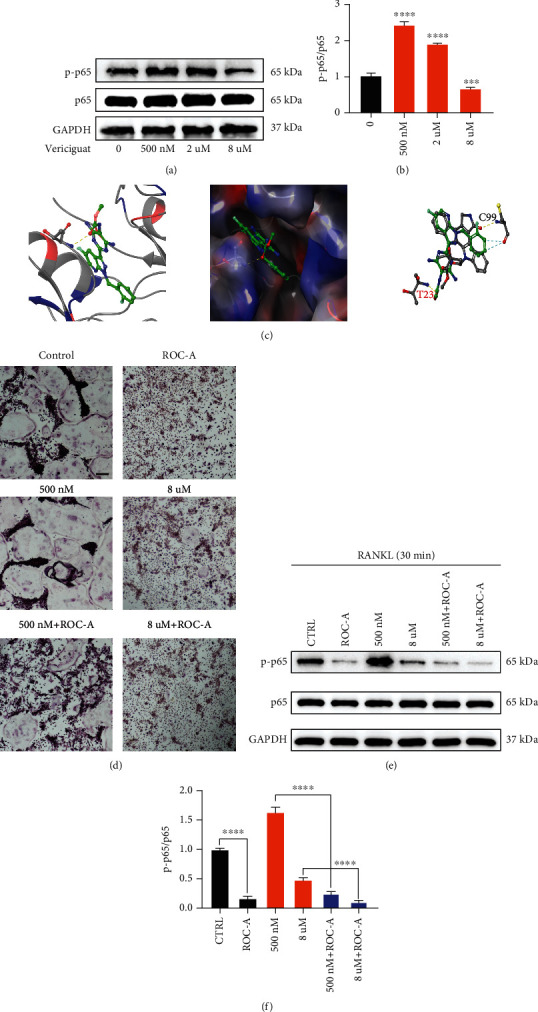
Dual effects of Vericiguat on osteoclast differentiation and bone resorption via a balance between VASP and NF-*κ*B. (a, b) The expression of NF-*κ*B protein in BMMs treated with Vericiguat only (*n* = 3). ∗ indicated a comparison with the control group (Vericiguat, 0 nM). (c) (A) The predicted optimal binding manner of Vericiguat at the ATP binding site of IKK*β* protein; hydrogen bonds (yellow lines). (B) MOLCAD representation showed the molecular lipophilic potential surface upon the bioactive pose of Vericiguat at the ATP binding site of IKK*β*. The blue represented hydrophilic, red represented lipophilic, and gray represented neutral moiety. Hydrogen bonds were displayed as yellow lines. (C) The overlap analysis of Vericiguat and the ligand of IKK*β* and the participating amino acid residue (Thr23) were marked. (d) TRAP staining of RANKL-induced BMMs treated with Vericiguat with or without the NF-*κ*B inhibitor ROC-A. (e, f) The expression of NF-*κ*B in RANKL-induced BMMs treated with Vericiguat with or without the NF-*κ*B inhibitor ROC-A. Scale bar = 500 *μ*m.

However, the inhibitory effect of high-dose Vericiguat on OC differentiation remains unclear. Further, we explored the expression of the NF-*κ*B signaling cascade in BMMs treated with Vericiguat only and found that Vericiguat could also dually regulate the activation of NF-*κ*B without MCSF and RANKL (Figures 7(a) and 7(b)). Regarding the repressive effect of Vericiguat on OC differentiation at high concentration, we deduced that high-dose Vericiguat may directly disturb the activation of the NF-*κ*B signaling cascade. Therefore, we introduced molecular modeling experiments and found that Vericiguat possessed similar molecular structure to the ligand of IKK*β* and could directly bind to the ATP binding pocket of the IKK*β* kinase domain function via hydrogen bond. However, the binding site was Thr23, but not the activated site, Cys99, which may be the reason for the inhibitory effect of Vericiguat on OC differentiation at high concentration (Figure 7(c)). In addition, blocking the activation of p65 could also disturb the promotive effect of Vericiguat on OC differentiation and enhance the suppressive effect of Vericiguat on OC differentiation (Figures 7(e) and 7(f)). Here, we hypothesized that Vericiguat may regulate OC differentiation via VASP/NF-*κ*B signaling crosstalk.

**Figure 8:**
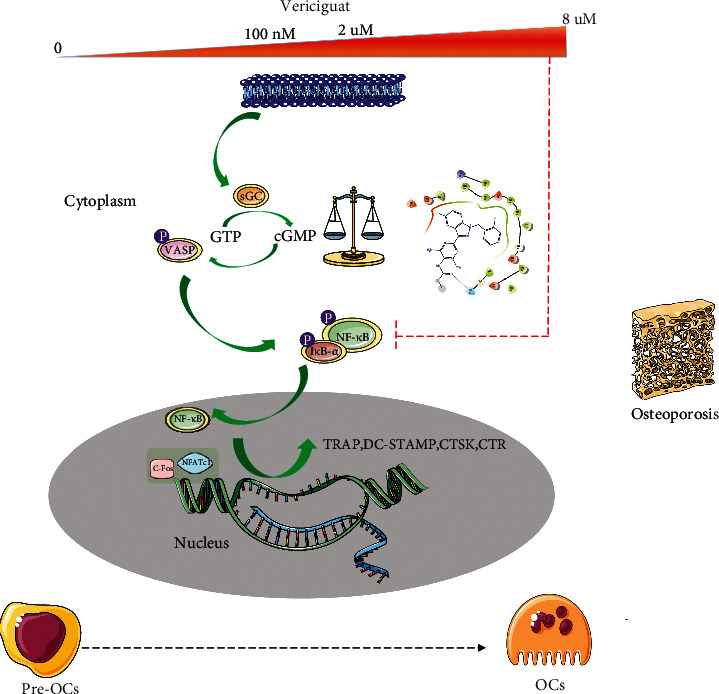
Dual effect of Vericiguat on RANKL-induced osteoclastogenesis and molecular mechanism. There may be a potential balance between the I*κ*B-*α*/NF-*κ*B signaling pathway and sGC/cGMP/VASP in BMMs. Vericiguat at low doses (0-2 *μ*mol/l) enhanced RANKL-induced osteoclastogenesis dominantly through upregulation of the VASP/I*κ*B-*α*/NF-*κ*B signaling pathway, whereas Vericiguat at high doses (4 or 8 *μ*mol/L) suppressed RANKL-induced osteoclastogenesis through directly inhibiting the I*κ*B-*α*/NF-*κ*B signaling pathway signaling cascade, which could disturb VASP-mediated activation of the I*κ*B-*α*/NF-*κ*B signaling pathway.

Taken together, at low concentration, Vericiguat dominantly showed promotive effect on OC differentiation via the VASP/NF-*κ*B signaling axis, whereas at high concentration, the increased binding of Vericiguat to IKK*β* kinase would result in the deactivation of p65 and inhibit OC differentiation (Figure 8).

### 4.8. Validation of the Effects of Vericiguat on OVX-Induced Bone Loss In Vivo

**Figure 9:**
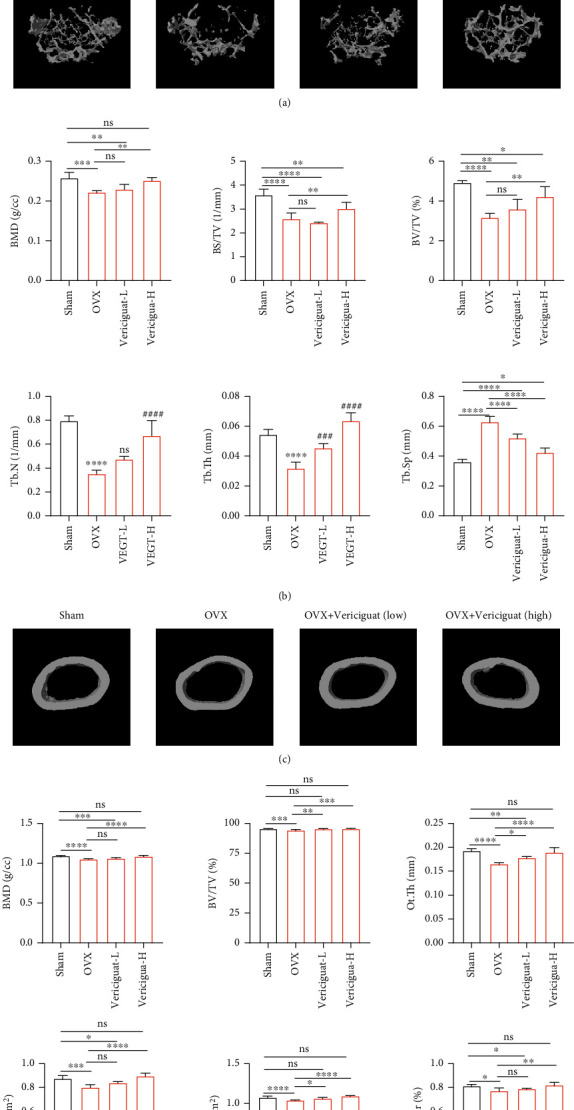
Validation of the effects of Vericiguat on OVX-induced bone loss in vivo indicated by microstructure analysis. (a) Representative 3-dimensional images of *μ*CT for mouse femur from the sham group (mock operation with DMSO injection), OVX group (OVX with DMSO injection), low-dose group (OVX with low-dose Vericiguat, 5 *μ*g/kg/day), and high-dose group (OVX with high-dose Vericiguat, 10 *μ*g/kg/day) (*n* = 5). (b) Quantitative results of bone structural parameters, including trabecular bone mineral density (BMD, g/cc), bone surface area/total volume (BS/TV; %), bone volume/total volume (BV/TV; mm^−1^), trabecular number (Tb.N; mm^−1^), trabecular spacing (Tb.Sp; mm), and trabecular thickness (Tb.Th; mm) within the selected metaphyseal region (*n* = 5). (c) Representative 3-dimensional images of *μ*CT for the mouse femur (cortical bone) from different groups above. (d) Quantitative results of bone structural parameters, including cortical bone mineral density (BMD; g/cc), bone volume/total volume (BV/TV; mm^−1^), total cross-sectional area inside the periosteal envelope (Tt.Ar; mm^2^), cortical bone area (Ct.Ar; mm^2^), cortical area fraction (Ct.Ar/Tt. Ar; %), and average cortical thickness (Ct.Th; mm). ∗ indicated a comparison with the sham group: ^∗∗∗^*p* < 0.001 and ^∗∗∗∗^*p* < 0.0001. # indicated a comparison between the VEGT-L/H group and OVX group at the same concentration of Vericiguat: ^##^*p* < 0.01, ^###^*p* < 0.001, and ^####^*p* < 0.0001. ns: not statistically significant.

As shown in Figure 9, the micro-CT results of BMD, BS/BV, BV/TV, Tb.N, Tb.Th, and Tb.Sp suggested that OVX mice exhibited obvious loss of trabecular bone, whereas this effect was restored by Vericiguat administration, either low or high dose (Figures 9(a) and 9(b)). However, the promotive effect of low-concentration Vericiguat on OC differentiation in vitro was not consistent with results in vivo. We deduced that there may be two reasons. Firstly, the low and high concentrations of Vericiguat in vitro cannot be considered equal to low and high doses in vivo. Secondly, cinaciguat, the same kind of Vericiguat, has been reported to promote OB differentiation and improve OVX-mediated bone loss [11]. Therefore, the proosteogenic effect of Vericiguat may in part offset the probony resorption at low concentration. Analysis of cortical bone parameters, BMD, BV/TV, Ct.th, Ct.Ar, Th.Ar, and Ct.Ar/Th.Ar, showed that OVX decreased the amount of cortical bone, whereas Vericiguat, especially at high doses, ameliorates the effect caused by OVX compared to the OVX group and sham group (Figures 9(c) and 9(d)). Collectively, the bioeffects of Vericiguat in vitro cannot be equal to those in vivo due to complex body environment.

**Figure 10:**
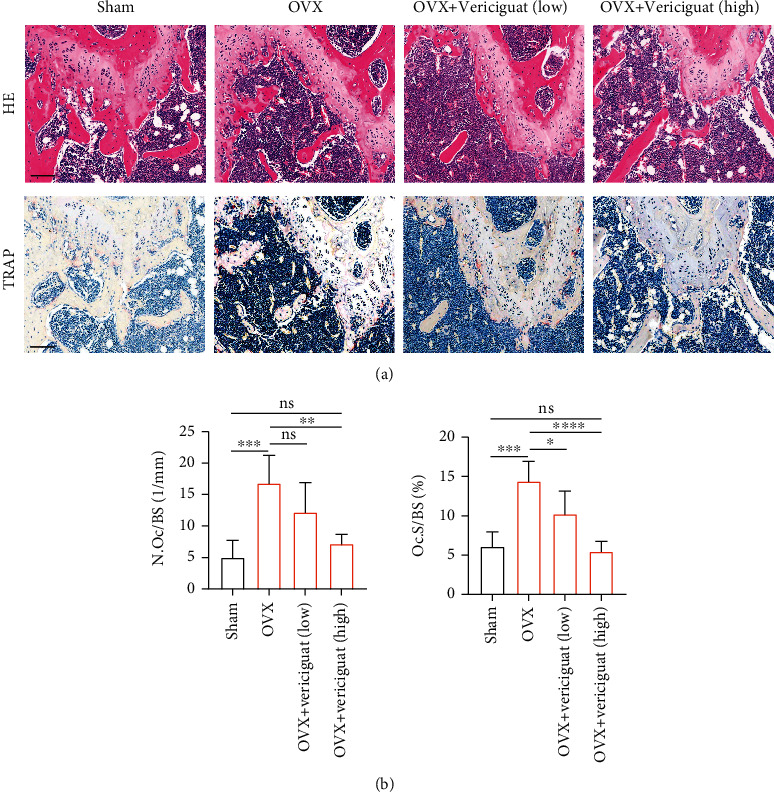
Validation of the effects of Vericiguat on OVX-induced bone loss in vivo indicated by histological analysis. (a) Representative images of H&E staining and TRAP staining within the selected metaphyseal region from the sham group, OVX group, low-dose group, and high-dose group (*n* = 5). (b) The number and area of TRAP^+^ cells per bone surface were quantified. ^∗^*p* < 0.05, ^∗∗^*p* < 0.01, ^∗∗∗^*p* < 0.001, and ^∗∗∗∗^*p* < 0.0001. Scale bar = 100 *μ*m.

As to histological analysis, H&E staining of the collected femurs suggested that the area of trabecular bone was markedly reduced in OVX mice, whereas mice treated with Vericiguat showed marked improvement of the trabecular bone area (Figure 10(a)). TRAP staining showed obvious enhancement in the total number and area of TRAP^+^ OCs along the trabecular bone in OVX mice, whereas Vericiguat decreased the area and the number of TRAP^+^ OCs per bone surface area (Figure 10(b)).

## 5. Discussion

Excessive activation of OCs may contribute to various OC-related diseases, such as rheumatoid arthritis and osteoporosis [19–22]. Thus, timely medical therapy targeting OCs is necessary for treating OC-related diseases [23, 24]. This present study firstly showed that Vericiguat dually regulated OC differentiation and bony resorption in mouse BMMs via bidirectionally affecting the activation of the I*κ*B-*α*/NF-*κ*B signaling pathway and nuclear translocation of NFATc1 in a dose-dependent manner. In fact, there are plentiful agents reported to possess dual effects according to concentration [25]. For OCs, baicalin has been reported to exert dual effects on OC formation in a concentration-dependent manner [26]. Compounds with dual effects have important clinical applications. Consequently, analyzing the effects of Vericiguat on the differentiation of OCs may deepen our understanding of the relationship between the pharmacological effects and dosage of Vericiguat on bone metabolism and also provide some medical references for future clinical application of Vericiguat to cardiovascular diseases.

The NO/GC/cGMP signaling cascade has been widely reported in regulating bone metabolism, and GC plays a significantly critical role [27, 28]. However, there exists controversy regarding the effect of NO/GC/cGMP on OCs [27–30]. Yaroslavskiy et al. ever reported NO-stimulated OC motility at low concentration, whereas at high concentrations, NO caused OC detachment and terminated resorption. And this effect was accomplished via the GC/cGMP/VASP cascade [9]. Another study from Joshua's team reported that cinaciguat (BAY 58–2667), a prototype of direct sGC activators, could reverse OVX-induced osteocyte apoptosis as efficiently as estradiol and promoted bone formation in vivo [11]. However, they found that the antiosteoporosis by cinaciguat was associated with enhancing function of OB, but not with the changes of OCs [11]. Nevertheless, Homer et al. found that oral administration of the sGC agonist could increase the number of OCs and bone resorption in the axial skeleton of Sprague-Dawley rats [31]. In fact, after careful studying the figures presented in Joshua's research, we found the decreased tendency regarding the number of OCs and eroded surface in the OVX^+^ cinaciguat group compared to the OVX group, although they demonstrated no statistical difference. Firstly, we deduced that the dose used in their mouse model (10 *μ*g/kg/day) may not reach the key point. In fact, the dose could be 10 mg/kg/day in animals [32]. Secondly, cinaciguat can be orally absorbed, but they chose ip injection. Thirdly, they did not perform the in vitro experiment regarding OCs. In addition, the abnormally high standard deviation regarding the number of OCs and eroded surface may also affect the real statistical results. Therefore, the exact mechanism of SC activators on OCs needs further investigation. In this present in vitro study, we found that Vericiguat could enhance RANKL-induced formation of functional OC under low concentration, whereas this effect was converted to inhibitory effect when BMMs were cocultured with Vericiguat at high concentration. What is more, Vericiguat also had dually regulatory effects on the RANKL-induced bone-resorbing ability. Collectively, these results suggested that Vericiguat dually regulated OC differentiation and bony resorption in mouse BMMs.

After RANKL binding to its receptor RANK on pre-OCs, RANKL would activate nuclear receptor NFATc1 and c-Fos [7]. NFATc1 has been one of the critical transcriptional regulators for RANKL-mediated OC differentiation [33]. After activation, NFATc1 will translocate into the nucleus and induce the expression of OC-specific genes, including DC-STAMP, TRAP, CTSK, and CTR [4, 34]. Our study suggested that Vericiguat dually regulated RANKL-induced expression of NFATc1 and its nuclear translocation during OC differentiation. In addition, the similar tendency regarding the mRNA expression changes of OC-specific genes (DC-STAMP, TRAP, CTSK, and CTR) also confirmed the bidirectional regulatory effects of Vericiguat on osteoclastogenesis. During osteoclastogenesis, c-Fos is also an essential mediator for osteoclastogenesis [4, 34]. Meanwhile, c-Fos is also involved in regulating the partial function of NFATc1 [4, 34]. In this present study, Vericiguat showed similar regulatory effects on RANKL-triggered expression of c-Fos under both mRNA and protein levels. Taken together, the results above indicated the dual effects of Vericiguat on RANKL-induced mature OC formation by regulating the transcriptional activity of c-Fos and NFATc1 and downstream critical OC-related gene expression.

During OC differentiation, NF-*κ*B, MAPK (ERK, p38, and JNK), and AKT signaling pathways are critically activated after RANKL stimulation [7]. Interestingly, in this present study, we found that Vericiguat dually regulated the phosphorylation of NF-*κ*B and the nuclear translocation of p65 in a dosage-dependent manner, without affecting MAPK and AKT signaling pathways. sGC activators could increase the amount pf sGC, which would excessively induce the activation of the VASP [18]. To further expound the potential regulatory mechanism through which Vericiguat regulated the NF-*κ*B pathway, we found increasing expression of VASP induced by either Vericiguat only or RANKL in a dose-dependent manner. Furthermore, in vitro experiment suggested that VASP was essential to OC differentiation, consistent with a recent study from Hu et al. [35]. Additionally, we found that Vericiguat only could also dually regulate the activation of NF-*κ*B. However, the promotive effect of Vericiguat on the expression of p-p65 was blocked by knocking down VASP, indicating that VASP may participate in OC differentiation via increasing the activation of the NF-*κ*B signaling cascade. However, with the increasing dose of Vericiguat imposed on BMMs, this promotive effect was missing. The results of molecular modeling experiments confirmed our hypothesis that high-dose Vericiguat may directly disturb the activation of the NF-*κ*B signaling cascade. In fact, Flores-Costa et al. reported that another sGC stimulator, praliciguat, could also reduce the phosphorylation of I*κ*B and NF-*κ*B to exert anti-inflammatory effect [35]. We deduced that Vericiguat may dominantly activate the sGC/VASP/I*κ*B-*α*/NF-*κ*B signaling pathway at low concentration. Nevertheless, with the increasing dose imposed on BMMs, Vericiguat would directly bind to IKK*β* to suppress nuclear factor-*κ*B (NF-*κ*B) activity. Thus, a delight balance between the I*κ*B-*α*/NF-*κ*B signaling pathway and sGC/cGMP/VASP may exist.

Despite these promising results above, there are several limitations in this present study. Firstly, this present study demonstrated the dual effects of Vericiguat on osteoclastogenesis, which was inconsistent with most of previous studies that NO/sGC/cGMP inhibits OC differentiation and maturation. We proposed a delight balance between the I*κ*B-*α*/NF-*κ*B signaling pathway and sGC/cGMP/VASP. At low concentration, the enhanced effect of RANKL-induced osteoclastogenesis by Vericiguat may be associated with the activated VASP/I*κ*B-*α*/NF-*κ*B signaling pathway. However, with the increasing concentration of Vericiguat, the direct inhibition of the I*κ*B-*α*/NF-*κ*B signaling pathway would dominantly counteract the promoting effect of VASP on the I*κ*B-*α*/NF-*κ*B signaling pathway and suppress osteoclastogenesis. However, the exact mechanism of Vericiguat on OC differentiation needs further investigation. Secondly, bone loss in vivo is a complex biological process related to various factors, such as estrogen deficiency, inflammatory condition, or aging [36–38]. We only used one model in this present study and confirmed the protective effect of Vericiguat on OVX-induced bone loss at a high dose. However, the promotive OC differentiation of low-concentration Vericiguat in vitro was not validated in vivo. Therefore, more bone loss model in vivo experiment will be required to confirm the dual effects of Vericiguat on bone loss, such as the LPS-induced inflammatory bone loss model. Thirdly, OBs also play a critical role in bone metabolism. Therefore, the biological effects of Vericiguat on OBs in vitro and in vivo also need to be explored, although other sGC activator, such as cinaciguat, has been reported to increase the proliferation, differentiation, and survival of OBs [11]. Our study will further focus on the effect of Vericiguat on OB differentiation both in vivo and in vitro.

In conclusion, our present study demonstrated the dual effects of Vericiguat on the formation of functional OCs in a concentration-dependent manner. The regulatory effect of Vericiguat on OCs was achieved by the bidirectional activation of the I*κ*B-*α*/NF-*κ*B signaling pathway, and a potential balance between the I*κ*B-*α*/NF-*κ*B signaling pathway and sGC/cGMP/VASP may exist. However, the exact mechanism of Vericiguat on OC differentiation both in vitro and in vivo needs further investigation.

## Figures and Tables

**Table 1:** Primers used for quantitative PCR.

Targeted gene	Forward (5′-3′)	Reverse (3′-5′)
GAPDH	TGACCACAGTCCATGCCATC	GACGGACACATTGGGGGTAG
c-Fos	CCAGTCAAGAGCATCAGCAA	AAGTAGTGCAGCCCGGAGTA
NFATc1	CCGTTGCTTCCAGAAAATAACA	TGTGGGATGTGAACTCGGAA
TRAP	CTGGAGTGCACGATGCCAGCGACA	TCCGTGCTCGGCGATGGACCAGA
CTR	TGGTTGAGGTTGTGCCCA	CTCGTGGGTTTGCCTCATC
CTSK	CTTCCAATACGTGCAGCAGA	TCTTCAGGGCTTTCTCGTTC
DC-STAMP	TCCTCCATGAACAAACAGTTCCAA	AGACGTGGTTTAGGAATGCAGCTC
VASP	GTGCGGAAGGAGCTACAGA	AGGCAGGGAAAGCAGGT

## Data Availability

Data will be available when requested.
